# Detection of Direct Oral Anticoagulants in Patient Urine Samples by Prototype and Commercial Test Strips for DOACs – A Systematic Review and Meta-analysis

**DOI:** 10.1055/s-0041-1732437

**Published:** 2021-09-24

**Authors:** Andrea Martini, Job Harenberg, Rupert Bauersachs, Jan Beyer-Westendorf, Mark Crowther, Jonathan Douxfils, Ismail Elalamy, Christel Weiss, Svetlana Hetjens

**Affiliations:** 1 Department of Medical Statistics and Biomathematics, Medical Faculty Mannheim, Ruprecht-Karls-University Heidelberg, Mannheim, Germany; 2 Ruprecht-Karls-University, Heidelberg, Germany; 3 DOASENSE GmbH, Heidelberg, Germany; 4 Department of Vascular Medicine, Klinikum Darmstadt GmbH, Darmstadt, Germany; 5 Thrombosis Research Unit, Department of Medicine I, Division Hematology, University Hospital “Carl Gustav Carus” Dresden, Dresden, Germany; 6 Department of Medicine, McMaster University and Thrombosis and Atherosclerosis Research Institute, Hamilton, Ontario, Canada; 7 Department of Pharmacy, Namur Thrombosis and Hemostasis Centre (NTHC), Namur Research Institute for Life Sciences (NARILIS), University of Namur, Namur, Belgium; 8 Qualiblood sa, Namur, Belgium; 9 Hematology and Thrombosis Centre, Hôpital Tenon, INSERM U938, Sorbonne Université, AP-HP, France; 10 I M Sechenov First Moscow State Medical University, Department of Obstetrics and Gynecology, Russia

**Keywords:** meta-analysis, DOAC Dipstick, direct oral anticoagulants, point-of-care test

## Abstract

The DOAC Dipstick accurately detects the presence or absence of factor Xa (DXI) and thrombin inhibitor (DTI) classes of direct oral anticoagulants (DOACs) in patients' urine samples on DOAC treatment. The aim of the study was to systematically review the literature and compare the performance of prototype and commercial test strips with a meta-analysis.

A systematic literature search of electronic databases PubMed (MEDLINE) and Cochrane Library was performed. Heterogeneity between studies was calculated using the Chi-squared test and the I ^2^ index. A random effects model was used to pool data to compare the performance of prototype and commercial test strips.

Using PRISMA reporting guidelines, four of 1,081 publications were eligible for inclusion in the meta-analysis: three reporting on prototype (DXI *n* = 658, DTI *n* = 586) and one on commercial test strips (DXI *n* = 451, DTI *n* = 429). Sensitivity and specificity of DXI and DTI detection did not differ significantly between the prototype and commercial test strips. Odds ratios were 0.718 and 0.365 for sensitivity and 1.211 and 1.072 for specificity of DXI and DTI (p-values between 0.3334 and 1.000), respectively. The pooled sensitivity and specificity values for DXI were 0.968 ( *p* = 0.1290, I ^2^ 47.1%) and 0.979 ( *p* = 0.1965, I ^2^ 35.9%), and for DTI 0.993 ( *p* = 0.1870, I ^2^ 37.5%) and 0.993 ( *p* = 0.7380, I ^2^ 0%), respectively.

Prototype and commercial DOAC test strips did not differ in their ability to detect DXI and DTI in patient urine samples. This supports the confidence in use of the DOAC Dipstick test, although it needs to be validated in specific patient populations.

## Introduction

The number of patients with non-valvular atrial fibrillation and thromboembolic events is increasing, mainly because the population is aging. 1 2 Direct oral anticoagulants (DOACs) are preferred over vitamin-K antagonists for preventing stroke in patients with non-valvular atrial fibrillation and for preventing and treating venous thromboembolism 3 4 because DOACs cause fewer intracranial bleeds, have fewer interactions with food and other drugs, and have a faster onset and offset of action. 5 Furthermore, regular drug monitoring is not required with DOACs because of their more predictable pharmacodynamic and pharmacokinetic properties. 6

DOACs can be detected in the laboratory by measuring the activated thromboplastin time and prothrombin time, although this depends on drug levels and reagent's sensitivity. Further tests include thrombin-specific clotting assays, such as the diluted thrombin time test and the ecarin clotting time. The viscoelastic hemostatic methods using thromboelastography is another test. DOACs can also be measured using chromogenic substrates and liquid chromatography tandem mass spectrometry (LC-MS/MS). 6 However, in emergency situations (such as bleeding, urgent surgery or thrombolysis), a fast, accessible and accurate point-of-care (POC) test is needed for detecting DOACs. 7

A qualitative POC DOAC Dipstick test was developed to detect DOACs in the urine. This was possible because 35–80% of dabigatran, apixaban, edoxaban and rivaroxaban are excreted unchanged into the urine. 8 Differences between the two types of test strips were that prototype test strips determine DXI and DTI on separate strips for analysis in separate urine samples while the commercial DOAC Dipstick (DOASENSE GmbH, Heidelberg, Germany) has separate pads for DXI and DTI on one test strip. Material and the technique for immobilization of reagents on pads differed between the types of test strips. The medium for color identification by the observer was urine sample and surface of the pad of DOAC Dipstick for prototype and commercial versions of test strips, respectively. The correctness of investigator's interpretation of the color was performed by trained laboratory personal 9 and liquid-chromatography mass spectrometry (LC-MS/MS) 10 11 for protype and commercial test strips, respectively. These differences could lead to different performance characteristics such as sensitivity, specificity, accuracy, negative and positive predictive values of the two types of test strips.

The aim of the investigation was to summarize available data in the literature on test strips performance to see whether the test can confidently be used in clinical practice. We systematically searched literature databases for studies investigating the detection of DXI and DTI in patient urine samples using test strips and conducted a meta-analysis to compare the performance of prototype and commercial type of test strips. In addition, based on the results of our meta-analysis we performed simulations to investigate the predictive values in populations with a lower proportion of DOAC intake.

## Methods

### Search Strategy

A systematic literature search was performed between 1993 up to October 2020 to identify relevant studies in PubMed (MEDLINE) and Cochrane Library databases. One additional abstract 12 was found in the Wiley Online Library. The literature search was performed in collaboration with librarians at the University of Heidelberg. The reference lists of all included papers were hand-searched to identify other relevant articles. The search string is listed in Table 1 .

**Table 1:** Search string for the meta-analysis

(“direct oral anticoagulant*”[tiab] OR doac*[tiab] OR”new oral anticoagulant*”[tiab] OR Noac*[tiab] OR”Dabigatran”[mh] OR “Rivaroxaban”[mh] OR “apixaban”[nm] OR “Rivaroxaban”[nm] OR “Dabigatran”[nm] OR “edoxaban” [nm] OR “apixaban”[tiab] OR “Rivaroxaban”[tiab] OR “Dabigatran”[tiab] OR “edoxaban” [tiab]) AND (“Point-of-Care Testing”[Mesh] OR Plasma[Mesh] OR Serum[Mesh] OR Urine[Mesh] OR “Point of care”[tiab] OR Plasma[tiab] OR Serum[tiab] OR Urine[tiab] OR Dipstick*[tiab] OR “Blood Coagulation Tests”[Mesh] OR “Coagulation”[tiab] OR “Mass Spectrometry”[Mesh] OR “Mass Spectrometry”[tiab] OR “International Normalized Ratio*”[tiab] OR INR[tiab] OR “Partial Thromboplastin Time”[tiab] OR aptt[tiab] OR Ptt[tiab] OR “Prothrombin Time*”[tiab] OR Pt[tiab] OR Thromboelastography[tiab] OR Thromboelastometry[tiab] OR “Thrombin Time*”[tiab] OR “whole blood clotting”[tiab] OR “chromogenic”[tiab] OR Hemoclot[tiab]) AND (“sensitivity and specificity”[Mesh] OR sensitiv*[tiab] OR “predictive value*”[tiab] OR accurac*[tiab] OR diagnosis[Subheading:noexp] OR diagnos*[tiab] OR specificity[tiab])

Inclusion criteria were the determination of DOACs in urine samples of patients treated with rivaroxaban, apixaban, edoxaban and dabigatran. Duplicate publications, narrative reviews, case reports, and studies that measured DOACs using coagulation and chromogenic tests, chromatography methods or blood based POC tests were excluded. Studies were screened and eligible studies were identified by one researcher (AM) and confirmed by another (SH). Any discrepancies were resolved by discussion. Each included study was evaluated for risk of bias using the checklist of methodological quality assessment using the QUADAS-2 method. 13 Risk of bias was assessed for patient selection, index test, reference standard, flow and timing. Applicability was granted for patient selection, index test, and reference standard. The risk of bias was considered low if all two or three categories were fulfilled, as high if one of the categories was unfulfilled, and unclear if more than one category were not fulfilled, respectively. All studies independent of their risk of bias were included in the analysis. 14

### Statistical Analysis

Statistical analyses were performed using SAS software, release 9.4 (Cary, USA) and MetaDiSc software, release 1.4 (Madrid, Spain). 15 The qualitative data of the prototype and commercial test strips (true positive, true negative, false positive, and false negative values) were presented for rivaroxaban, apixaban, edoxaban, and dabigatran and stratified by study ( Table 2 ).

**Table 2:** Data used for pooled analysis and meta-analysis

	DOAC	Type of test strip	True positive	False positive	True negative	False negative
Study 1 20	Rivaroxaban	Prototype	449	8	395	16
	Dabigatran		480	4	476	0
Study 2 21	Rivaroxaban	Prototype	77	2	50	1
	Apixaban		64	3	62	1
	Dabigatran		76	0	52	1
Study 3 22	Rivaroxaban	Prototype	24	0	29	0
	Apixaban		26	0	29	0
	Dabigatran		29	0	29	0
Study 4 11	Rivaroxaban	Commercial	147	8	421	3
	Apixaban		160			10
	Edoxaban		127			4
	Dabigatran		427	3	448	2
	*DXI total*	*Prototype, commercial*	*1074*	*21*	*986*	*35*
	*DTI total*	*Prototype, commercial*	*1012*	*7*	*1005*	*3*

The sensitivity is defined as the proportion of true positive results in relation to the population treated with a DOAC (factor Xa or thrombin inhibitor) and the specificity as the proportion of true negative results in relation to the population of untreated controls (not treated with a factor Xa or thrombin inhibitor). Sensitivity and specificity of individual and pooled studies were analyzed using MetaDiSc. The sensitivities and specificities of prototype and commercial test strips were compared using Chi-squared test. If the presumptions of the Chi-squared test were not fulfilled, Fisher's exact test was used alternatively. Test results were considered as statistically significant at p-values below 0.05. Furthermore, odds ratios (OR) and 95% CI of sensitivity and specificity derived from the two types of test strips were considered as relative frequencies (not normally distributed data) and compared by Chi-squared test or Fisher's exact test as appropriate. Heterogeneity between studies was calculated using chi-squared heterogeneity test and the I ^2^ index at a p-value of < 0.05. An I ^2^ index value gauges heterogeneity - between 0 to 25% indicates insignificant heterogeneity; > 25% to 50% low heterogeneity; > 50% to 75% moderate heterogeneity; and > 75% high heterogeneity. 14 The random effects model according to DerSimonian and Laird was used to analyze pooled data 16 –this technique takes any heterogeneity between the studies into account. Forest plots were created for sensitivity and specificity of studies showing weight by size of points and in percent, values with 95% CI, I ^2^ index and p-values for differences. 17 The area under the curve (AUC) of the summary receiver operating characteristic (SROC) curve was calculated to assess the diagnostic accuracy of the meta-analysis. 18 Based on the sensitivity and specificity analyses, the accuracy, negative predictive value (NPV) and positive predictive value (PPV) of test strips results were calculated for the simulated prevalences of 1%, 10%, 30% and 60% based on Bayes' rule. The simulated prevalence represents the simulated proportion of a population who take DOACs in a given period of time.

The study was conducted according to the Preferred Reporting Items for Systematic Reviews and Meta-Analyses (PRISMA) statement. 19

## Results

### Identified Studies

**Fig. 1:**
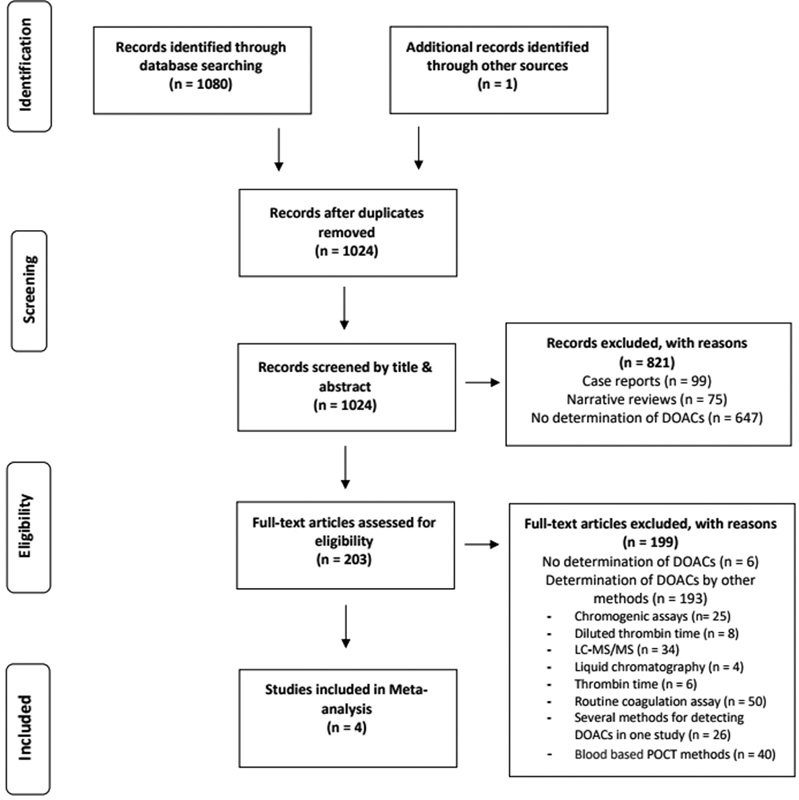
PRISMA flow chart for selection of included studies.

1,081 potentially eligible studies were found in the database search. After removing duplicate publications, narrative reviews, case reports, studies that did not detect DOACs, and studies that did not detect DOACs in urine samples of patients treated with DOACs, four studies were eligible for inclusion in the meta-analysis. The PRISMA flow chart shows the exclusion and inclusion of studies ( Fig. 1 ).

Three studies used the prototype test strips. Study 1 was a single-center study ( *n* = 465 rivaroxaban, *n* = 480 dabigatran). 20 Study 2 was an international collaborative study performed with urine samples of patients treated with rivaroxaban ( *n* = 78), apixaban ( *n* = 65), and dabigatran ( *n* = 77) including the results of day one of two days of testing by participating centers. 21 Study 3 evaluated urine samples of patients treated with rivaroxaban ( *n* = 24), apixaban ( *n* = 26), and dabigatran ( *n* = 29) and of controls not treated with anticoagulants ( *n* = 29) 22 of which preliminary results were reported. 12 Results of a positive or negative adjudication of colors of factor Xa and thrombin inhibitor pads of the test strips by observers were compared with those of trained laboratory personal. Study 4 used the commercial DOAC Dipstick in a multicentre trial using urine samples of patients treated with rivaroxaban ( *n* = 150), apixaban ( *n* = 170), edoxaban ( *n* = 131), and dabigatran ( *n* = 429) ( Table 2 ). In this study, the thrombin inhibitor pad of the DOAC Dipstick served as a negative control if patients were treated with a DXI and vice versa. Therefore, a control group not taking an anticoagulant was not required. Results of the visual adjudication of colors of factor Xa and thrombin inhibitor pads of DOAC Dipstick by observers were analyzed following dichotomization of the quantitative results of DOACs in urine at a cut-off value of <30 ng/ml DOAC determined liquid chromatography mass spectrometry. 11 As reported, participants of all four studies were on stable treatment with DOACs and were admitted to outpatient care units. All patients had a diagnosis of non-valvular atrial fibrillation or venous thromboembolism. DOACs were given orally at doses of 10 mg od, 15 mg od, or 20 mg od (rivaroxaban), 2.5 mg and 5 mg bid (apixaban), 30 mg and 60 mg od (edoxaban), and 110 mg and 150 mg bid (dabigatran). Urine samples were collected at random time between 1.5 hour and 24 hour after administration of the immediate prior dose. Patients had to have normal renal function for treatment with a DOAC and had stable health conditions when investigated. Creatinine clearance was not determined in patients. 11 20 21 22

### Risk of Bias

Study quality was evaluated using QUADAS-2 (Quality Assessment of Diagnostic Accuracy Studies 2), a standardized tool for quality assessment of studies of diagnostic accuracy. All studies were open label. All patients had diagnoses of non-valvular atrial fibrillation and venous thromboembolism. They were included consecutively for studies 1, 3, and 4 (low risk of bias). Study 2 used samples of selected patients (unclear risk). The index test was compared in studies 1, 2 and 3 to visual analysis of test pads by laboratory trained personal (low risk) and in study 4 to LC-MS/MS (low risk). The flow and timing of the index test and reference standard was performed the same day (studies 1 and 3, low risk) and was variable for studies 2 and 4. However, DOACs stored at -24°C in urine samples are stable over 24 months ( https://doasense.de/files/ENGLISH_IFU_DOASENSE-Control-Urines_DOASENSE-WI7-5-8-EN-Rev02.pdf ) resulting in an adjudication of low risk of bias. Applicability assessment was the same as for study quality ( Table 3 ).

**Table 3:** Risk of bias of the trials assessed by Quandas-2 as a standardized tool for quality assessment of studies of diagnostic accuracy

Study, Reference	Risk of bias				Applicability concerns		
	Patient selection	Index test	Reference standard	Flow and timing	Patient selection	Index test	Reference standard
Study 1 20	low	low	low	low	low	low	low
Study 2 21	unclear	low	low	unclear	unclear	low	low
Study 3 22	low	low	low	low	low	low	low
Study 4 11	low	low	low	low	low	low	low

### Comparison of Prototype and Commercial Test Strips

The results of the individual studies were summarized as true positive, false positive, true negative and false negative detection of factor Xa and thrombin inhibitors in urine of patients treated with the DXIs rivaroxaban, apixaban and edoxaban and the DTI dabigatran ( Table 2 ).

The sensitivity, specificity and accuracy ranged between 0.941 and 0.998 at all simulated prevalences (1%, 10%, 30% and 60%) for all DOACs and both prototype and commercial test strips. The PPV decreased with decreasing prevalence as expected for DOACs. In contrast, the NPV increased up to 0.999 for all DOACs and both types of test strips with decreasing prevalence. No clinically relevant differences were found between prototype and commercial test strips ( Table 4 ).

**Table 4:** Simulated comparison of sensitivity, specificity, PPV, NPV and accuracy for the prototype and commercial test strips at a prevalence between 1% and 60%

		Prototype test strip				Commercial test strip			
	Prevalence	1%	10%	30%	60%	1%	10%	30%	60%
DXI	Sensitivity	0.973				0.962			
	Specificity	0.978				0.981			
	PPV	0.304	0.828	0.949	0.985	0.343	0.851	0.957	0.987
	NPV	0.999	0.997	0.988	0.960	0.999	0.996	0.984	0.946
	Accuracy	0.978	0.977	0.976	0.975	0.981	0.979	0.976	0.970
DTI	Sensitivity	0.998				0.995			
	Specificity	0.993				0.993			
	PPV	0.586	0.940	0.984	0.995	0.602	0.943	0.985	0.996
	NPV	0.999	0.999	0.999	0.997	0.999	0.999	0.998	0.993
	Accuracy	0.993	0.993	0.994	0.996	0.993	0.993	0.994	0.996
Rivaroxaban	Sensitivity	0.970				0.980			
	Specificity	0.979				0.981			
	PPV	0.322	0.839	0.953	0.986	0.347	0.854	0.957	0.987
	NPV	0.999	0.996	0.987	0.956	0.999	0.998	0.991	0.970
	Accuracy	0.979	0.978	0.977	0.974	0.981	0.981	0.981	0.981
Apixaban	Sensitivity	0.989				0.941			
	Specificity	0.968				0.981			
	PPV	0.238	0.775	0.930	0.979	0.338	0.849	0.956	0.987
	NPV	0.999	0.999	0.995	0.983	0.999	0.993	0.975	0.918
	Accuracy	0.968	0.970	0.974	0.981	0.981	0.977	0.969	0.957

The frequencies of correct positive, correct negative, false positive and false negative detection of factor Xa and thrombin inhibitors using prototype and commercial test strips are shown in Table 5 . The specificity of pooled data on DXI and DTI detection by prototype and commercial test strips was not significantly different. The sensitivity of prototype and commercial version of test strips was also not significantly different for DXI and DTI ( Table 5 ).

**Table 5:** Sensitivity and specificity data: Frequencies of correct positive, correct negative, false positive and false negative results by pooled data using prototype and commercial test strips to detect DXI and DTI

		Prototype test strip	Commercial test strip	Total	*p* Value
**Sensitivity**					
DXI	True positive	640	434	1074	0.3334*
	False negative	18	17	35	
DTI	True positive	585	427	1012	0.5768°
	False negative	1	2	3	
Specificity					
DXI	True negative	565	421	986	0.6730*
	False positive	13	8	21	
DTI	True negative	557	448	1005	1.0000°
	False positive	4	3	7	

*= Chi-squared test, °= Fisher's Exact test

The sensitivity and specificity of the pooled data from prototype and commercial test strips were also not significantly different for DXIs and DTI, with values between 0.962 and 0.998 (p-values between 0.3334 and 1.0000). The ORs of sensitivity were higher for DXI and DTI tests using prototype test strips and the ORs of specificity were higher for DXI and DTI of the commercial test trips (all not significant), respectively ( Table 6 ). The sub-analysis for rivaroxaban and apixaban also revealed high sensitivity and specificity values between 0.941 and 0.989 for both types of test strips and ORs were all not significantly different ( Table 6 ). Edoxaban could not be evaluated because data were only available from study 4. 11

**Table 6:** Sensitivity, specificity and OR with 95%CIs for detection of DXIs, DTI, rivaroxaban and apixaban by prototype and commercial test strips

		Prototype test strip value (95% CI)	Commercial test strip value (95% CI)	OR (95% CI)	*p* Value
DXI	Sensitivity	0.973 (0.957; 0.984)	0.962 (0.940; 0.978)	0.718 (0.366; 1.409)	0.3334*
	Specificity	0.978 (0.962; 0.988)	0.981 (0.972; 0.988)	1.211 (0.497; 2.948)	0.6730*
DTI	Sensitivity	0.998 (0.991; 1.000)	0.995 (0.983; 0.999)	0.365 (0.033; 4.038)	0.5768°
	Specificity	0.993 (0.982; 0.998)	0.993 (0.981; 0.999)	1.072 (0.239; 4.816)	1.0000°
Rivaroxaban	Sensitivity	0.970 (0.952; 0.982)	0.980 (0.943; 0.996)	1.515 (0.438; 5.238)	0.7800°
	Specificity	0.979 (0.962; 0.990)	0.981 (0.964; 0.992)	1.110 (0.434; 2.839)	0.8271*
Apixaban	Sensitivity	0.989 (0.940; 1.000)	0.941 (0.895; 0.971)	0.178 (0.022; 1.411)	0.1033°
	Specificity	0.968 (0.910; 0.993)	0.981 (0.964; 0.992)	1.735 (0.452; 6.666)	0.4254°

*= Chi-squared test, °= Fisher's Exact test.

An OR > 1 indicates that DOAC Dipstick has a higher sensitivity or specificity compared with prototype test strip.

### Meta-analysis

**Fig. 2:**
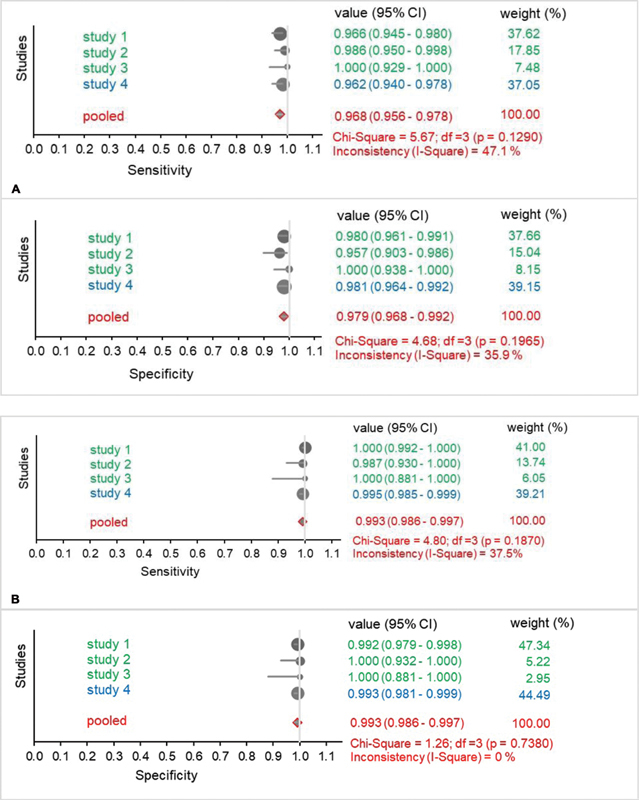
Forest plot showing sensitivity (upper panel) and specificity (lower panel) analysis results for studies with DXI ( Figure 2A ), DTI ( Figure 2B ), rivaroxaban ( Figure 2C ), and apixaban ( Figure 2D ) using the prototype (green) and commercial (blue) test strips. Pooled data are shown in red. Values are presented with 95% CI. Size of the circles represents the weight of the studies. P-values were determined using the Chi-squared test. Inconsistency/Heterogeneity of studies is shown in %. (A) Forest plots DXI, sensitivity (upper panel), specificity (lower panel). (B) Forest plots DTI, sensitivity (upper panel), specificity (lower panel). **(C)** Forest plots rivaroxaban, sensitivity (upper panel), specificity (lower panel). (D) Forest plots apixaban, sensitivity (upper panel), specificity (lower panel).

The forest plots in Fig. 2 show the sensitivity and specificity of DXI and DTI (dabigatran, rivaroxaban, and apixaban) detection in four studies. The sensitivity and specificity were 0.968 and 0.979 for DXI detection, respectively ( Fig. 2A ) and both 0.993 for DTI ( Fig. 2B ). The sub-analysis of rivaroxaban ( Fig. 2C ) and apixaban ( Fig. 2D ) data showed sensitivity and specificity values between 0.958 and 0.980. Inconsistency values ranged from 0% to 58.3%.

**Figure:**
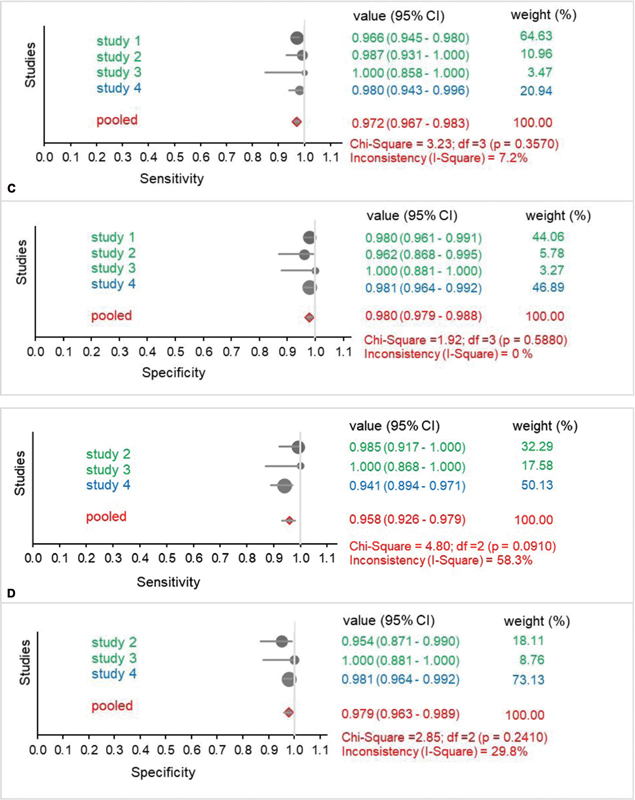


The area under the curve (AUC) of summary receiver operating characteristic (SROC) curve analysis showed AUC values of 0.9957 for DXI, 0.9990 for DTI dabigatran, 0.9964 for rivaroxaban, and 0.9947 for apixaban. Edoxaban data were only available for the commercial DOAC Dipstick test so are not reported.

## Discussion

The present analysis demonstrates that the small-scale laboratory prototype and the large-scale produced commercial test strips have comparable performances despite the multiple differences of productions techniques and methods to prove the correctness of the visual color assessment of the observers. Moreover, the meta-analysis results quantified the evidence of diagnostic accuracy for the DXI and DTI with its sensitivity and specificity from commercial DOAC Dipstick results in existing studies. Accordingly, the results of the meta-analysis should increase the confidence in the validity of DOAC Dipstick to qualitatively detect DOACs in urine samples of patients treated with apixaban, edoxaban, rivaroxaban, and dabigatran. 11

Other POCT tests able to determine the presence of DOACs from blood samples have also been reported in the literature. Thromboelastographic methods, 23 global coagulation assays 24 , specific coagulation POCT testing methods using ecarin reagent for determination of dabigatran 25 , a dielectric microsensor after recalcification of a small amount whole blood sample 26 and others. 27 The performance of the urine based DOAC Dipstick to identify DOACs on the studied populations was at least as high as those obtained obtained with specific testing from patients' plasma and whole blood samples using chromogenic assays for DXI or tests based on ecarin reagent for dabigatran measurements.

The results of this meta-analysis confirm these high sensitivity and specificity of the prototype and commercial test strips and thereby increases the confidence in the results of the DOAC Dipstick test- supporting their use in emergency care medicine and other medical conditions where rapid medical decision-making processes are required. 11 27 Importantly, test results need to be interpreted only in connection with the patient's clinical situation. Examples for clinical indications are patients with acute ischemic stroke to help in thrombolysis or mechanical decision-making, acute or haemorrhagic for deciding to use an antidote, before an acute major urgent surgical intervention, before epidural anesthesia, to confirm stopping of a DOAC before a required temporary interruption or to check adherence to therapy. 11

The comparative simulated prevalence analysis confirmed that the prototype and commercial test strips have comparable performances with no clinically relevant differences. Measures of sensitivity and specificity indicate the quality of a diagnostic test, but the question still remains whether DOACs are really present in a patient's system. In these situations, predictive values are important since they indicate the magnitude to which the test result can be relied on to rule out clinically important concentrations of drug. The PPV and NPV represent the proportions of positive or negative test results that were identified correctly. However, these values depend on the fraction of people evaluated who are taking a DOAC at the time of analysis. In a clinical context, the PPV should be interpreted with caution because if only a few patients are taking a DOAC, the PPV will be lower than was seen in this study. The PPV also depends on the prevalence in a specific clinical context. In the studies included in our meta-analysis, outpatients with stable DOAC therapy were included – in “real world use” the PPV may vary by disease (such as infections, malignancy, emergency care, major operations, thromboembolism and major bleeding, among others).

In contrast, the NPV increased with decreasing prevalence, indicating a high accuracy of the test strips and suggesting a higher probability of no DOACs being present in a patient's system with increasing numbers of patients being analyzed. This is likely unless DOAC excretion into the urine is reduced by nephropathy, or if food, drugs and drug metabolites change the urine's color. 28 29 30 31 In these circumstances, the creatinine pad and urine color pad are important controls for accurate DOAC Dipstick results. 11 32

In this analysis, we combined the results of four studies in one random-effect meta-analysis. In contrast to the fixed effects model, the random effects model assesses both intra- and inter-study variance and provides wider confidence intervals and a better estimate of the effect size. 33 The meta-analysis showed a pooled sensitivity and specificity of >96% for DXIs (rivaroxaban, apixaban and edoxaban) and DTI (dabigatran). Regarding the accuracy of both types of test strips, the area under the curve values of the SROC were 0.9957 for DXIs and 0.9990 for DTIs. In addition, the overall heterogeneity of sensitivity and specificity values for all DOACs were categorized as low (between 0% to 58.3%) according Higgins et al. 14 By combining the four eligible studies in one meta-analysis, this analysis provides evidence for the generalizability of study results using the commercial version of test strips.

Limitations of the present analysis need to be considered. All studies were performed under the guidance of the same investigator. However, the tests were evaluated by different participants in the studies which corresponds to the clinical application. The four included studies used not completely identical study designs and investigated different DOACs: apixaban was investigated in three of the four studies 11 21 22 and edoxaban in one study. 11 This means the exploratory power of edoxaban was low in the meta-analysis. However, the one study investigating edoxaban was a large-scale study 11 that provided enough data to evaluate the accuracy of edoxaban detection by the DOAC Dipstick. A further potential limitation was the heterogeneity between studies; however, we found heterogeneity to be low to moderate with values between 0% and 58% when compared with the literature. 34 Future studies should further investigate this heterogeneity using subgroup analysis, sensitivity analysis or meta-regression. 35

The DOAC Dipstick was indicated as a useful tool for detecting DOACs in emergency care by the NICE Guidance document. 36 Furthermore, the DOAC Dipstick was mentioned in a Guideline document for the treatment of the femoral fracture as a suitable on-site test in patients with acute hip-fracture on handling further anticoagulant medication. 37 Several investigator-initiated studies are ongoing in acute major orthopaedic surgery, in patients with ischemic stroke to support rapid medical decision processes and adherence to their therapy for validation of DOAC Dipstick test. They include investigations of plasma levels of rivaroxaban, edoxaban, apixaban and dabigatran compared with qualitative DOAC Dipstick results in more heterogenous patient populations. The DOAC Dipstick may also be used to manage patients who need to be switched from DOAC therapy immediately to low-molecular weight heparin upon admission to hospital such as upon hospitalization for COVID-19. 38

In conclusion, this study shows the robustness of the DOAC Dipstick in detecting DOACs in patient urine samples, thereby increase the confidence that this test is suitable for use in clinical practice. The analysis highlighted the high accuracy of the DOAC Dipstick in detecting rivaroxaban and apixaban. Simulation of prevalence analysis showed the NPV is very high, which is important when intake of DOACs is unknown. Further studies are ongoing to validate the DOAC Dipstick in various clinical emergency situations and to compare DOAC levels quantified in blood with DOAC levels detected qualitatively in urine samples.

## Notes

**What is Known about the Topic?**

- Rapid and accurate determination of oral direct factor Xa (DXI) and thrombin inhibitors (DTI) remains challenging in emergency medical situations.
- Several studies have been published on the detection of DXI and DTI in patient urine samples by prototype and commercial test strips.
- Differences between the two types of test strips include that DOAC Dipstick test determines DXI and DTI on one strip only, materials and techniques for immobilization of reagents and medium for color identification.

**What Does this Paper Add?**

- No statistically significant differences were found for sensitivity and specificity between both versions of test strips increasing the confidence in the high performance characteristics of the commercial version of DOAC Dipstick.
- In this systematic review and meta-analysis, both included types of test strips demonstrated an over 96% sensitivity and specificity for detecting DXI and DTI in a single test only from patients' urine samples.
- The simulation of the prevalence showed the very high negative predictive value that is important in a general population and in specific patients when intake of DOACs in unknown.
